# Symbiodiniaceae Community Structure and Thermal Tolerance in Soft Corals from Captive Aquarium Environments

**DOI:** 10.1093/icb/icag042

**Published:** 2026-05-14

**Authors:** Nia S Walker, Henry Zucco, Eridaly Basave, Jie Yi Denise Chen, Rylie Crow, Evelyn Chavez-Gonzalez

**Affiliations:** Kravis Department of Integrated Sciences, Claremont McKenna College, Claremont, CA 91711, USA; Kravis Department of Integrated Sciences, Claremont McKenna College, Claremont, CA 91711, USA; Kravis Department of Integrated Sciences, Claremont McKenna College, Claremont, CA 91711, USA; Kravis Department of Integrated Sciences, Claremont McKenna College, Claremont, CA 91711, USA; Kravis Department of Integrated Sciences, Claremont McKenna College, Claremont, CA 91711, USA; Kravis Department of Integrated Sciences, Claremont McKenna College, Claremont, CA 91711, USA

## Abstract

Corals in aquarium systems experience environmental conditions that differ substantially from those in the wild, potentially altering their microbiomes and influencing health and stress resilience. In this study, we investigated shifts in symbiotic dinoflagellates (family Symbiodiniaceae) in common soft corals sourced from aquarium stores across Southern California. Using ITS2 amplicon sequencing, we characterized symbiont communities across coral genera and store locations. We then conducted a high heat pulse assay on a subset of samples to examine relationships between thermal tolerance, symbiont community composition, coral genus, and source location. We found that Symbiodiniaceae communities were highly similar among corals within shared aquarium store environments, rather than exhibiting genus-specific symbiont profiles, and most corals had *Cladocopium*-dominated symbiont communities. Thermal tolerance varied strongly among coral genera, with *Briareum* (green star polyps) displaying the highest heat tolerance. These findings suggest that captive aquarium environments can structure coral symbiont communities across distantly related hosts although the coral host strongly influences physiological responses to heat stress. Understanding how artificial environments shape coral holobionts is relevant not only for the aquarium trade but also for coral husbandry in research and conservation, where symbiont composition can influence survival and experimental outcomes.

## Introduction

Coral reefs are among the most biologically valuable and vulnerable marine ecosystems on Earth (Hughes et al. 2017). Reef-building stony corals (order Scleractinia) construct the calcium carbonate frameworks that support ~25% of marine biodiversity (Sobha et al. 2023). There has been significant research into reef-building coral resilience capacity and mitigation efforts as anthropogenic-caused stressors such as ocean warming, acidification, and pollution continue to cause immense damage to these ecosystems (Hoegh-Guldberg et al. 2017). Additionally, cnidarians such as soft corals (class Octocorallia) play critical ecological roles in shaping reef community dynamics through increasing habitat complexity, influencing local nutrient cycling, and occupying disrupted or degraded benthic spaces in many reef systems (Lalas et al. 2024; Pupier et al. 2024). Further, studies have found that soft coral cover can increase on reefs where hard coral abundance has declined (Inoue et al. 2013; Baum et al. 2016; Tkachenko et al. 2022; Lalas et al. 2024). This highlights the increasing importance of understanding soft coral physiology to help predict the future trajectory of coral reef ecosystems and how they may respond to environmental and community composition shifts.

Like reef-building corals, soft corals function as holobionts composed of the coral host and associated microbial communities, and these interactions are central to the coral host’s health, resilience, and ecological performance (Woo et al. 2017). Soft and hard corals also share fundamentally similar physiological traits, including highly integrated mutualisms with family Symbiodiniaceae dinoflagellates (Mohamed et al. 2023), mucus production (Goulet et al. 2020), innate immune pathways (Mansfield & Gilmore 2019), and associations with diverse bacterial, archaeal, and viral communities (McCauley et al. 2023). These shared traits suggest that key mechanisms governing host-microbiome interactions may be conserved across cnidarian lineages (Villela et al. 2024). Soft corals, which often exhibit high fecundity (Teixidó et al. 2016), tissue regeneration (Nadir et al. 2023), and tolerance to variable husbandry conditions (Chang et al. 2020; Larkin et al. 2023), additionally offer practical advantages for experimental manipulation and replication. As such, they provide valuable opportunities to test hypotheses regarding functional responses in simplified, well-controlled systems that can inform broader coral holobiont research.

Soft corals also provide valuable systems for studying coral responses to thermal stress. While hard corals have been the primary focus of heat stress and bleaching research (Putnam et al. 2017; Drury 2020; Cornwell et al. 2021), a growing body of species-specific studies suggests that soft corals can exhibit stress responses that both overlap with and differ from those documented in scleractinian corals (Slattery et al. 2019; Kim et al. 2025; Mezger et al. 2025). The co-occurrence of hard and soft corals within shared reef habitats, and in aquarium systems, within close physical proximity, enables common-garden experimental designs that minimize environmental variability while allowing direct intergeneric comparisons. Such controlled experiments can help unravel conserved versus lineage-specific responses to thermal stress, identify mechanisms of resilience or vulnerability, and refine predictions of community-level responses to climate change. Incorporating soft corals alongside scleractinians, therefore, broadens the comparative framework necessary for developing a more integrated understanding of coral physiology under warming oceans. Additionally, the proximity between multiple genera in the same controlled tank presents a unique opportunity to study the influence of common garden environments on soft corals' physiological composition.

Studies have documented that soft corals, similar to reef-building hard corals, can harbor highly variable Symbiodiniaceae communities, including common *Cladocopium* spp. and *Durusdinium* spp., that significantly contribute to host energy budgets and resilience to thermal stress (Van Oppen et al. 2005; Sikorskaya et al. 2020; Maucieri & Baum 2021; Butler et al. 2026). Broader coral microbiome research indicates that Symbiodiniaceae communities can also shape bacterial community structure and stability, with implications for holobiont health across environments by influencing nutrient supply, protection against pathogens, and host physiological functions under environmental variability (Gervais et al. 2025). The Symbiodiniaceae family consists of highly taxonomically diverse genera (LaJeunesse et al. 2018), which contribute variably to holobiont nutrient exchange, metabolic stability, stress resilience, and immune regulation.

Symbiont communities can be disrupted due to acute and chronic stress, particularly high thermal stress as it will trigger a suite of physiological responses in corals that can result in the systematic loss of symbionts from coral host cells (Weis 2008; Helgoe et al. 2024; Tortorelli et al. 2025). There is relatively little known about soft coral thermal stress capacity, compared to the vast body of literature on reef-building hard corals (Pandolfi et al. 2011; Drury 2020; Hughes et al. 2021; Walker et al. 2024; Walker et al. 2025). Experimental exposure of the soft coral *Sinularia heterospiculata* to elevated temperatures has been shown to induce physiological disruption of the coral-dinoflagellate symbiosis, including loss of symbiont cells and alterations to host lipid metabolism (Sikorskaya et al. 2020). Controlled experiments examining the combined effects of increased temperature and light stress on the soft coral *Sarcophyton glaucum* showed that elevated temperatures significantly decreased photosynthetic efficiency, and that heat exacerbated photophysiological stress when coupled with high light (Travesso et al. 2023). Despite their ecological presence, common soft coral genera such as *Xenia, Briareum*, and *Sarcophyton* remain poorly characterized experimentally with respect to holobiont responses to thermal stress. As such, targeted investigation of these genera is necessary to determine how Symbiodiniaceae structure influences thermal responses in soft corals, and whether common trends described in scleractinians, such as association with thermal tolerance or fast growth (Cunning & Baker 2020; Wang et al. 2023; Liang et al. 2025), also extend to soft corals.

Despite their ecological importance, soft corals remain underrepresented in experimental studies of coral thermal stress and symbiont ecology (van de Water et al. 2018; Easson et al. 2024), particularly in controlled aquarium environments. Aquarium systems provide a unique opportunity to investigate coral holobiont dynamics in replicated common garden conditions, where multiple coral genera are maintained under shared environmental parameters. In this study, we investigated how aquarium store environments influence Symbiodiniaceae communities in three common aquarium soft coral genera (*Xenia, Briareum*, and *Sarcophyton*) across multiple locations in Southern California. Using ITS2 amplicon sequencing, we characterized symbiont community composition and tested whether symbiont structure was more strongly associated with coral host genus or with shared aquarium environments. We then evaluated thermal tolerance using a CBASS-style heat stress assay (Evensen et al. 2023) to examine relationships among symbiont composition, coral genus, and physiological stress responses. Understanding how captive conditions shape microbial composition and influence coral resilience under environmental stress may inform strategies to improve coral health in the aquarium trade and on coral reefs.

## Methods and Materials

### Sample Collection

We collected coral specimens representing the genera *Xenia* (pulse coral), *Briareum* (green star polyp), and *Sarcophyton* (toadstool leather) between June and November 2025 from six retail aquarium stores located throughout the Greater Los Angeles metropolitan area: Tropical Reef Store (2 June 2025, n = 8), Jan’s Tropical Fish (4 June 2025, n = 2), CK Fishworld (22 October 2025, n = 6), and Bob’s Tropical Fish (n = 4), Pisces Fish and Coral (n = 5), and Art’s Underwater Reef (n = 3) (8 November 2025). We took 2–4 ~2 cm tissue samples per colony, depending on size, and immediately preserved them in DNA/RNA Shield (Zymo Research). All tools used to clip tissues were cleaned between samples (10% bleach, 70% ethanol, and deionized water). We also measured light intensity (PAR) and water temperature at multiple locations within each aquarium tank, which showed minimal variation among stores at the time of sampling (Table S1), though these values may not reflect longer-term or dynamic tank conditions. Together, these measurements and samples provide a snapshot of the biological material and associated environmental conditions across all sampled systems (Table S1). All coral samples were transported on ice to Claremont McKenna College and stored at −80°C. Upon completion of our sampling we had 10 green star polyps (GSP), 10 leather corals (LC), and 8 pulse corals (PC) (Table S1). As we did not genotype host tissue and relied on common names from suppliers, we classified corals to the genus level (*Xenia, Sarcophyton*, and *Briareum*).

For Tropical Reef Store, we sampled pulse corals from a single tank that we conservatively treated as one colony; however, the store manager was unsure whether the pulse corals represented one genotype. Additionally, there is some debate in the literature regarding the correct taxonomic designation for green star polyps concerning whether *Briareum* or *Pachylvaria* are a single or sister taxa (Samimi-Namin & van Ofwegen 2016). We classified green star polyps as *Briareum* spp. in this study.

### Thermal stress assay

Coral samples from Jan’s Tropical Fish (Pulse = 2) and Tropical Reef Store (Pulse = 1, Leather Coral = 4, and Green Star Polyp = 3) were acclimated for one week at 25°C prior to the heat stress experiment, chosen to approximate typical tank temperatures across stores and to serve as the experimental baseline. Tanks were outfitted with overhead LED lights with day (100–300 PAR)/night (0–5 PAR) settings, heaters, temperature probe, and air pump. We also checked light intensity, temperature, and salinity (30–35 ppt) daily (Table S2). Replicates from each colony were divided between one control tank and two heated tanks. We used a modified CBASS-style heat pulse ramp assay (Evensen et al. 2023) to quickly reveal variation in thermal tolerance. The assay ran from June 11 10:00 AM to June 17 10:00 AM, 2025. The ramp profile was as follows: 10:00 AM–1:00 PM ramp from 25°C baseline to peak temperature, hold at peak temperature 1:00 PM–4:00 PM, ramp down to the baseline 4:00 PM–5:00 PM, then hold at baseline until 10:00 AM. Peak temperature began at 30°C then increased by 2°C every two days until we reached 34°C (Fig. 3A). Corals from Jan’s Tropical Fish entered the thermal stress assay on June 12 10:00 AM and remained in the assay for five days, because they were collected one day later than Tropical Reef Store corals (Fig. 3A).

We measured mortality, bleaching, and polyp visibility daily at 10:00 AM, 1:00 PM, 4:00 PM, and 5:00 PM, in heated and control samples. Specifically, we categorically measured mortality and bleaching as none, partial, or total for each sample. Polyp visibility was also qualitatively measured: no visibility (N: None), <25% visible (M: Minimal), and >25% visible (Y: Yes). For pulse corals, which always have visible polyps, we measured whether their tentacles were extended and pulsing (N: closed, M: open but not pulsing, and Y: open and pulsing) and their polyp stalks were standing (N: fully drooped, M: slight droop, Y: fully upright). All measurement categories were converted into scores from one to three then cumulatively added to create a health score that ranged from one (total mortality meant no scores in bleaching and polyp categories) to nine (threes from each category). For the pulse coral polyp visibility score, we averaged the tentacle and stalk scores. These scoring approaches were adapted to account for inherent differences in morphology and behavior among coral genera, which may contribute some variation across taxa. We also sacrificed tissue samples from all living controls and heated samples at the end of the thermal stress assay (June 17, 2025 at 10:00 AM), which were immediately preserved in DNA/RNA shield (Zymo).

### Evaluation of Symbiodiniaceae communities

We extracted genomic coral holobiont DNA using the Quick-DNA/RNA™ Microprep Plus Kit, following manufacturer protocol (Zymo Research). We targeted the ribosomal internal transcribed spacer 2 (ITS2) region using the SYM_VAR_5.8S2 (5′-TCG TCG GCA GCG TCA GAT GTG TAT AAG AGA CAG GAA TTG CAG AAC TCC GTG AAC C-3′) (Hume et al. 2015) and SYM_VAR_REV (5′-GTC TCG TGG GCT CGG AGA TGT GTA TAA GAG ACA GCG GGT TCW CTT GTY TGA CTT CAT GC-3′) (Hume et al. 2013), which were modified to include Illumina forward and reverse sequences (underlined). We conducted PCR amplification with Hotstart 2x Mastermix and using the following protocol: initial denaturation at 95°C for 3 min; 30 cycles of 95°C for 30 s, 55°C for 30 s, and 72°C for 30 s; and a final extension at 72°C for 5 min. We cleaned ITS2 amplified DNA with Mag-Bind TotalPure NGS beads, following the manufacturer’s protocol (OMEGA Bio-Tek). We conducted a second round of PCR to attach unique Nextera XT Indexes (Set A), using the same PCR protocol but with 25 cycles. After cleaning again with magnetic beads, we pooled all samples into a DNA library for sequencing on the Illumina MiSeq v3 platform with 2 × 250 paired-end reads (University of California at Riverside, Institute for Integrative Genome Biology). We then used a local Symbiodiniaceae reference database from SymPortal (Hume et al. 2019) to identify symbiont variation at the genus, species, and population levels.

### Statistical analyses

We conducted all statistical analyses in RStudio v20206.01.1 (Posit 2025). We performed a Fisher’s exact test and post-hoc pairwise Fisher’s test to determine whether there was a relationship between *Cladocopium*-dominated colonies and aquarium stores or coral genera. Symbiodiniaceae alpha diversity (Shannon diversity index) was calculated using the *vegan* package (Oksanen et al. 2007). Principal component analysis (PCA) was performed on Symbiodiniaceae relative abundance data using the rda function in the *vegan* package, and loadings (*Cladocopium* vs. *Durusdinium* dominance) were used to interpret variation along principal components. We performed Kruskal–Wallis tests to examine the relationships between (1) symbiodiniaceae alpha diversity and store, (2) symbiodiniaceae alpha diversity and coral genera, (3) heated—control samples’ *Durusdinium* proportion difference at the end of the thermal stress assay and coral genera, (4) coral genera and heated samples’ health score on the final thermal stress assay day, and (5) dominant symbiont (*Cladocopium* or *Durusdinium*) and heated samples’ health score. We also conducted post-hoc Dunn’s tests with Benjamini–Hochberg correction (FDR = 0.05) on pairwise comparisons from Kruskal–Wallis analyses. We used Wilcoxon signed-rank tests to determine whether there was a significant increase in the *Durusdinium* proportion between control and heated samples, whether control and heated samples’ health scores were significantly different after the thermal stress assay, and to confirm the two experimental tanks did not significantly impact responses (Wilcoxon signed-rank test, *P* = 0.5879). All significant p-values were determined to be <0.05 in this study. Analyses were conducted using univariate and pairwise approaches due to the uneven structure of the sampling design across coral general and stores.

## Results

### Symbiodiniaceae community variation across aquarium environments

**Fig. 1:**
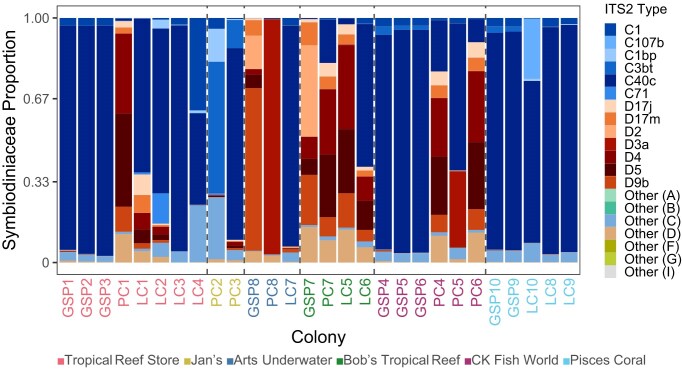
Bar plot of relative proportions of Symbiodiniaceae across coral colonies and aquarium stores. Values represent averages of replicate samples per colony. Aquarium stores are distinguished by color and vertical dashed line. Cooler colors represent *Cladocopium* (C) and warm colors represent *Durusdinium* (D). Individual colonies are colored by store: Pink: Tropical Reef Store (n = 8); Yellow: Jan’s (n = 2); Blue: Art’s Underwater (n = 3); Green: Bob’s Tropical Reef (n = 4); Purple: CK Fish World (n = 6); Cyan: Pisces Coral (n = 5).

Across all colonies, symbiont communities were overwhelmingly dominated by *Cladocopium* spp. and *Durusdinium* spp., with *Cladocopium* as the primary symbiont genus in the majority of samples (20/28 colonies) (Fig. 1). At finer taxonomic resolution, *Cladocopium* ITS2 types C40c and C1 and *Durusdinium* types D4 and D5 were the most prevalent across hosts and stores. Although seven Symbiodiniaceae genera were detected overall, non-*Cladocopium* and non-*Durusdinium* taxa (*Symbiodinium, Breviolum, Fugacium, Gerakladium, Clade I*) were rare and collectively contributed only ~0.02% of community composition (Fig. 1). Importantly, both *Cladocopium* and *Durusdinium* were detected in all colonies, indicating widespread background presence of these genera regardless of clear dominance patterns within each sample.

Patterns of symbiont dominance varied across coral genera. *Cladocopium* dominated 80% of green star polyps (GSP) across all stores (averaged proportion 99.23% ± 0.18% SE), and the remaining 20% of GSP were *Durusdinium*-dominated (99.10% ± 0.47%). Leather corals (LC) were similarly *Cladocopium*-dominated in 90% of colonies (90.42% ± 5.09%); two LC at Bob’s Tropical Reef (LC5 and LC6) were exceptions, harboring primarily *Durusdinium* (96.7%). Pulse corals (PC) showed the greatest compositional variability among genera, with 62.5% of colonies *Durusdinium*-dominated (88.57% ± 4.53%) and the remaining 37.5% *Cladocopium*-dominated (86.52% ± 9.76%). Notably, all PCs from Jan’s had *Cladocopium* proportions (96.3% ± 1.38%) (Fig. 1). Pisces Coral colonies had strongly homogenous *Cladocopium* communities across multiple genera (GSP, LC), with C40c as the dominant ITS2 type.

There was no overall significant difference in the proportion of *Cladocopium* versus *Durusdinium*-dominated colonies among stores (Fisher’s exact test, *P* = 0.065). However, the largely *Durusdinium*-dominated Bob’s Tropical Reef had significantly different Symbiodiniaceae dominance compared to *Cladocopium*-dominated Pisces Coral (pairwise fisher exact test, *P* = 0.048). Similarly, while there was no overall significant relationship between proportion of *Cladocopium*-dominance and coral genus (Fisher’s exact test, *P* = 0.063), pulse corals were significantly more *Durusdinium*-dominant than leather corals (pairwise fisher exact test, *P* = 0.043).

### Symbiodiniaceae alpha diversity

**Fig. 2:**
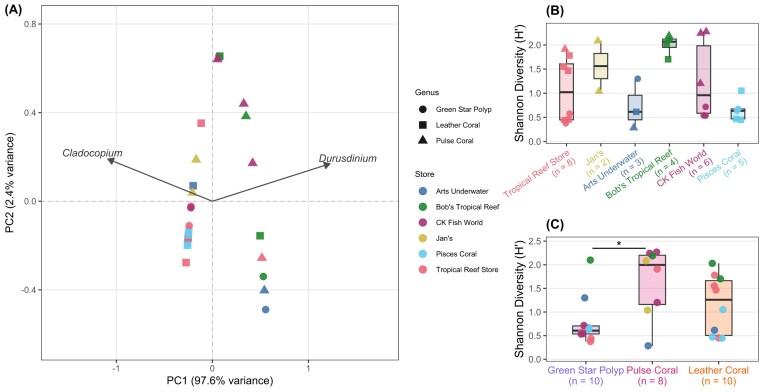
Symbiodiniaceae community composition diversity drivers. Aquarium stores are represented by color and coral genus is represented by point shape.(A) Principal component analysis of soft coral colonies’ Symbiodiniaceae ITS2 type-level community diversity. (B) Shannon diversity (H’) of Symbiodiniaceae communities grouped by aquarium stores. Each point represents a single coral colony. Note Jan’s (n = 2) and Art’s Underwater (n = 3) have insufficient replication for robust distributional inference. (C) Shannon diversity (H’) of Symbiodiniaceae communities across aquarium stores grouped by genus. The asterisk denotes *P* < 0.05 between Pulse Coral and Green Star Polyp shannon diversity (Kruskal–Wallis).

We found that differences between Symbiodiniaceae communities were largely driven by *Cladocopium* or *Durusdinium* dominance, explaining 97.6% of our principal component analysis (PC1) (Fig. 2A). Additionally, alpha diversity (richness and evenness of Symbiodiniaceae community composition) slightly varied within and among stores. Bob’s Tropical Reef exhibited the highest alpha diversity, with colonies across all three coral genera clustering tightly around H’ = 2.1. In contrast, Pisces Coral showed the lowest alpha diversity (median H’ = 0.62), with four of five colonies clustering between H’ = 0.45–0.65. Tropical Reef Store and CK Fish World both showed high within-store variance, with interquartile ranges approximately H’ = 0.45–1.60 and H’ = 0.60–1.98, respectively (Fig. 2B). Alpha diversity did not significantly differ among stores (Kruskal-Wallis, *P* = 0.10, post-hoc Dunn’s test all *P* > 0.05) or host genera (Kruskal-Wallis, *P* = 0.067). We did detect significantly higher alpha diversity in pulse corals (median H’ = 2.00) than green star polyps (median H’ = 0.606) (post-hoc Dunn’s test, *P* = 0.031, z = −2.32), while leather corals (median H’ = 1.26) did not differ significantly from either genus (Dunn’s test, both *P* > 0.10) (Fig. 2C).

### Thermal tolerance across coral genera and Symbiodiniaceae communities

**Fig. 3:**
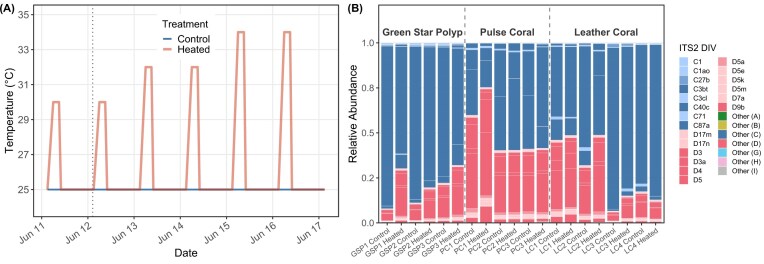
Thermal stress assay factors.(A) Thermal stress assay temperature profile with heated (red) and control (blue) lines. The heated line is an average of two experimental tanks and the control represents one control tank. The vertical dotted line represents when Jan’s corals were introduced into the thermal stress assay (6 instead of 7 days). (B) Stacked barplot of relative proportions of Symbiodiniaceae types by coral colony heated vs. control samples, averaged replicates. Samples originated solely from Tropical Reef Store (n = 8) and Jan’s (n = 2).

Although *Cladocopium* spp. remained the dominant Symbiodiniaceae genus in all heated and control samples except for in one colony (Pulse coral 1, PC1), the proportion of *Durusdinium* spp. increased in heated samples relative to paired control fragments within the same colony for all colonies except pulse coral 2 (PC2) and leather coral 4 (LC4) (Fig. 3B). Across colonies, *Durusdinium* spp. increased on average 7.37% ± 2.48%, and this paired increase was significant (Wilcoxon signed-rank test, *P* = 0.027). Green star polyps showed the greatest mean increase in *Durusdinium* spp. (12.65% ± 4.28%), followed by leather corals (6.37% ± 4.28%), while pulse corals showed minimal change (3.43% ± 3.86%) (Fig. 3B). However, these genus-level differences in the magnitude of control vs. heated *Durusdinium* change were not significant (Kruskal-Wallis, *P* = 0.55; post-hoc Dunn’s test all *P* > 0.05).

**Fig. 4:**
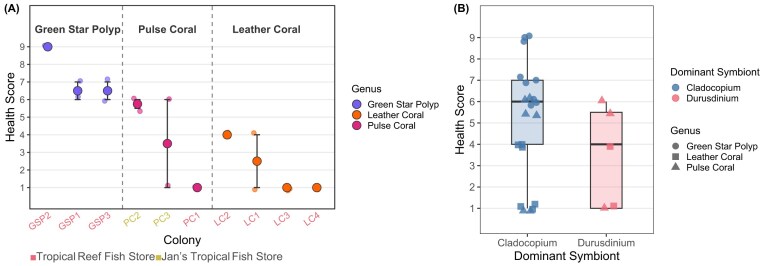
Thermal stress resilience (A) Health scores of soft coral colonies by genus (green star polyp, pulse coral, and leather coral) under heated treatment conditions at the final experimental timepoint (17 June 2025, 10:00:00 AM). *X*-axis label colors indicate store of origin: pink = Tropical Reef Store (n = 8); yellow = Jan’s (n = 2). (B) Health scores at the final timepoint by dominant Symbiodiniaceae genus, *Cladocopium* or *Durusdinium*. Shapes correspond to coral genus.

Heated corals had significantly lower health scores than their controls on the final day of the thermal assay (Paired Wilcoxon, *P* = 0.0046) (Fig. S1). Coral genera significantly predicted thermal tolerance (Kruskal-Wallis, *P* = 0.0015). Overall, heated green star polyps (GSP) exhibited significantly higher thermal tolerance than leather corals (Dunn’s test, *P* = 0.0011, z = 3.38) and pulse corals (Dunn’s test, *P* = 0.0023, z = 2.97), with an average health score of 7.33 ± 0.833. Heated leather corals (2.12 ± 0.642) and pulse corals (3.42 ± 1.37) had relatively low health scores and were thermally sensitive (Fig. 4A). There was no significant association between dominant Symbiodiniaceae genus (i.e., *Cladocopium* vs. *Durusdinium*) and heated coral health score (Kruskal–Wallis, *P* = 0.195) (Fig. 4B).

Although the sample sizes (Jan’s n = 2, Tropical Reef Store n = 8) were too small to investigate whether stores exhibited differential coral thermal tolerance, we did note that the lowest-scoring pulse coral originated from the Tropical Reef Store (its Day 6 health score = 1 out of 9), while the two higher scoring pulse corals came from Jan’s (their Day 6 average health score = 4.625 out of 9), suggesting some potential that store origin may contribute to thermal performance variation between conspecifics.

## Discussion

This study examined how captive aquarium environments may structure dinoflagellate symbiont (family Symbiodiniaceae) communities in soft corals and whether symbiont composition and coral genera relates to thermal stress responses. We sampled three common aquarium coral genera (*Briareum*—green star polyps, *Sarcophyton*—leather corals, and *Xenia*—pulse corals) from multiple Southern California aquarium stores.

We found that symbiont communities were dominated by *Cladocopium* and *Durusdinium* spp., with relatively little contribution from other Symbiodiniaceae lineages. Symbiont composition was broadly similar among corals maintained within the same aquarium environments, suggesting that shared husbandry conditions may shape symbiont assemblages across distantly related hosts. Thermal tolerance varied strongly among coral genera in our CBASS-style heat pulse assay. Green star polyps exhibited the highest thermal tolerance, maintaining relatively high health scores in the assay while leather and pulse corals were thermally sensitive. Overall, these results suggest that aquarium common garden environments can structure coral symbiont communities while host-specific traits may remain important drivers of thermal stress responses.

### Symbiodiniaceae community structure in captive aquarium environments

Despite detecting seven Symbiodiniaceae genera across all sampled colonies, symbiont communities were overwhelmingly dominated by *Cladocopium* (mostly C40c and C1) and *Durusdinium* (commonly D4 and D5). These two genera are commonly found in reef-building corals and soft corals (LaJeunesse et al. 2018; Goulet et al. 2019; Butler et al. 2023; Liang et al. 2025), and they may represent contrasting ecological strategies. *Cladocopium* spp. are often associated with faster coral host growth and higher productivity (Matsuda et al. 2023), although types C40c and C1 are variably tolerant lineages (van Oppen et al. 2001; Jones et al. 2008; Swain et al. 2017; Wang et al. 2023) in different reef and experimental systems. *Durusdinium* spp., including ITS2 types D4 and D5, are commonly linked to increased thermal tolerance in many scleractinian corals and may be present in more stressful or variable reef environments (Swain et al. 2017; Cunning & Baker 2020; Poquita-Du et al. 2020).

Our results found that distant soft coral genera tended to be dominated by *Cladocopium* spp. (71.4%), which suggests a strong preference for faster growing symbionts in relatively stable and constrained captive aquarium environments. This may reflect the influence of common environmental conditions, including light intensity, temperature stability, nutrient availability, and water chemistry, which are all typically tightly regulated in aquarium systems (Borneman 2008; Lehmann 2022). These factors can also influence symbiont competitiveness and host-symbiont compatibility in variable and hostile reef environments where certain symbiont lineages are favored across multiple coral hosts depending on environmental conditions (Patin et al. 2018; Klinges et al. 2023; Rola et al. 2025). Studies of soft corals, including *Sarcophyton, Xenia*, and *Briareum*, on reefs in the Indo-Pacific, the Red Sea, and Caribbean have similarly reported high *Cladocopium* proportions and have also found *Durusdinium, Symbiodinium*, and *Breviolum* (Van Oppen et al. 2005; Osman et al. 2020; Butler et al. 2026).

Based on our results, common garden conditions in aquaria facilitate symbiont homogenization across distant coral species. Previous studies have also observed in reef environments that some transplanted corals may converge on similar Symbiodiniaceae communities to native corals over time (Armstrong et al. 2024). Aquarium systems may also constrain the environmental symbiont pool available for uptake, limiting opportunities for hosts to acquire diverse symbionts from the surrounding water column (Lehmann 2022). In contrast, soft corals in natural reef environments may contain greater symbiont diversity that could fluctuate across seasons, years, and generations, which would facilitate the development of stronger host-symbiont specific fitness associations.

### Symbiodiniaceae alpha diversity

Overall Symbiodiniaceae alpha diversity was relatively low across sampled colonies, with most samples strongly dominated by few symbiont lineages. Low within-host symbiont diversity has been documented in other coral species, and in addition to a limited or homogeneous water environment could reflect competitive exclusion among symbionts within host tissues (LaJeunesse et al. 2003; Ng & Ang 2016; Howe-Kerr et al. 2020). In our dataset, variation in alpha diversity was largely driven by differences at the genus level between *Cladocopium* and *Durusdinium*. Among host genera, pulse corals harbored the highest symbiont diversity on average, while green star polyps showed consistently lower diversity largely due to their strong *Cladocopium* dominance. Alpha diversity variation within coral genera coming from different stores may reflect store-specific symbiont differences at the species or population levels that may be further illuminated in future studies with larger sample sizes and greater distances between aquarium locations.

### Thermal tolerance and Symbiodiniaceae dynamics

Our heat pulse stress assay revealed clear differences in thermal tolerance among coral genera. Green star polyps maintained relatively high health scores under elevated temperatures, while leather and pulse corals exhibited stronger signs of physiological stress including higher mortality, bleaching, and polyp/tentacle retraction. These results support previous literature that host-specific traits, beyond Symbiodiniaceae community, play an important role in determining thermal resilience in soft corals (Manzello et al. 2019; Drury 2020). There is limited previous literature on the thermal resilience capacity of leather corals (Chavanich et al. 2009), green star polyps (Ding et al. 2022), and pulse corals (Ziegler et al. 2014; Steinberg et al. 2021, 2022; Thobor et al. 2022). However, this is the first study to compare thermal tolerance capacity in these three soft coral genera and we found evidence that challenged these previous assumptions regarding thermal tolerance. This study highlights the importance of comparative thermal tolerance assays as researchers and policymakers work to identify and preserve thermal tolerance on coral reefs.

Thermal stress exposure was associated with a modest (7.37% average) but significant increase in the relative abundance of *Durusdinium* spp. symbionts across heated samples. Similar shifts toward *Durusdinium* dominance have been observed in scleractinian corals exposed to elevated temperatures and provide evidence of symbiont community restructuring toward more thermally tolerant symbionts in stressful environments (Cunning et al. 2015), although such patterns favoring thermally tolerant symbionts are less consistently documented in octocoral systems (Goulet & Coffroth 2003; Pelosi et al. 2021). Despite this shift, we did not detect a strong association between dominant symbiont genus and thermal tolerance in our assay. Colonies dominated by *Cladocopium* or *Durusdinium* performed similarly in the experiment, suggesting that host physiological traits may have exerted a strong influence on short-term stress responses. We did not measure coral host traits such as metabolic rates (Grottoli et al. 2006; Palardy et al. 2008; Parry et al. 2025) or genetic markers (Bellantuono et al. 2012; Kenkel et al. 2014; Kirk et al. 2018; Drury 2020), which could influence resilience to environmental stress.

### Conclusions and future directions

Understanding how captive environments shape coral holobionts has important implications for both the aquarium trade and experimental coral research. Aquarium systems represent common-garden environments where environmental conditions can strongly influence microbial community structure. These artificial environments may not reflect natural reef ecosystems but allow for assessing genetic diversity and resilience capacity in controlled and easily manipulable settings. These insights also provide a strong mechanistic framework for coral restoration initiatives, such as land-based nurseries, where understanding how controlled conditions influence holobiont assembly is essential for predicting the long-term success of outplanted corals (van Oppen et al. 2015; Schopmeyer et al. 2017; da Silva et al. 2019).

Our results suggest that aquarium environments can strongly influence Symbiodiniaceae community structure across multiple soft coral genera, promoting similar symbiont assemblages among coral hosts maintained under shared environmental conditions. Despite this convergence, thermal tolerance varied strongly among coral genera, indicating that host-specific physiological traits remain important determinants of stress responses. Broadly, these findings highlight the importance of considering both environmental context and host biology when evaluating coral symbioses and stress resilience in captive systems.

Study limitations include a relatively small sample size, particularly for the thermal stress assay, the narrow geographic focus of aquarium sampling, and the unknown environmental history of the coral specimens. Additionally, uneven sampling across coral genera and stores limited multivariate resolution of host, symbiont, and thermal tolerance relationships. Larger surveys across distant aquarium systems and coral species would help determine consistency and strength of the patterns observed in this study. Expanding to other components of the microbiome (e.g., 16S sequencing) may also reveal further complex patterns of variation and contributions to thermal stress capacity in this system.

## Supplementary Material

icag042_Supplemental_Files

## Data Availability

All data and R code can be retrieved on Github: https://github.com/niasymwalker/softcorals1/.

## Notes

From the symposium “Host-microbial interactions across biological scales in a changing world” presented at the annual meeting of the Society for Integrative and Comparative Biology, January 3-7th, 2026.
