# Supplementary material for: Symbiodiniaceae Community Structure and Thermal Tolerance in Soft Corals from Captive Aquarium Environments
Source: Integr Comp Biol. 2026 May 14;66:icag042. doi: 10.1093/icb/icag042 (PMC13196601; doi:10.1093/icb/icag042)
Supplement: icag042_Supplemental_Files [file icag042_supplemental_files.zip › FINAL 0104 supplementary captions.docx]

**Supplementary Materials**

**Table S1:** Summary of aquarium store environmental measurements and coral samples.

**Table S2:** Summary of thermal stress assay daily environmental measurements.

**Figure S1: Time series of mean health scores across the duration of the heat stress assay.** The dashed vertical line represents the point at which pulse corals from Jan’s were added into the experiment (6 instead of 7 days). Health scores represent the mean value of colonies in the experiment for a given timepoint. Dashed lines represent controls and solid lines represent heated, and error bars represent standard error.
