# Supplementary material for: Symbiodiniaceae Community Structure and Thermal Tolerance in Soft Corals from Captive Aquarium Environments
Source: Integr Comp Biol. 2026 May 14;66:icag042. doi: 10.1093/icb/icag042 (PMC13196601; doi:10.1093/icb/icag042)
Supplement: icag042_Supplemental_Files [file icag042_supplemental_files.zip › icb-2026-0104-File008.pdf]

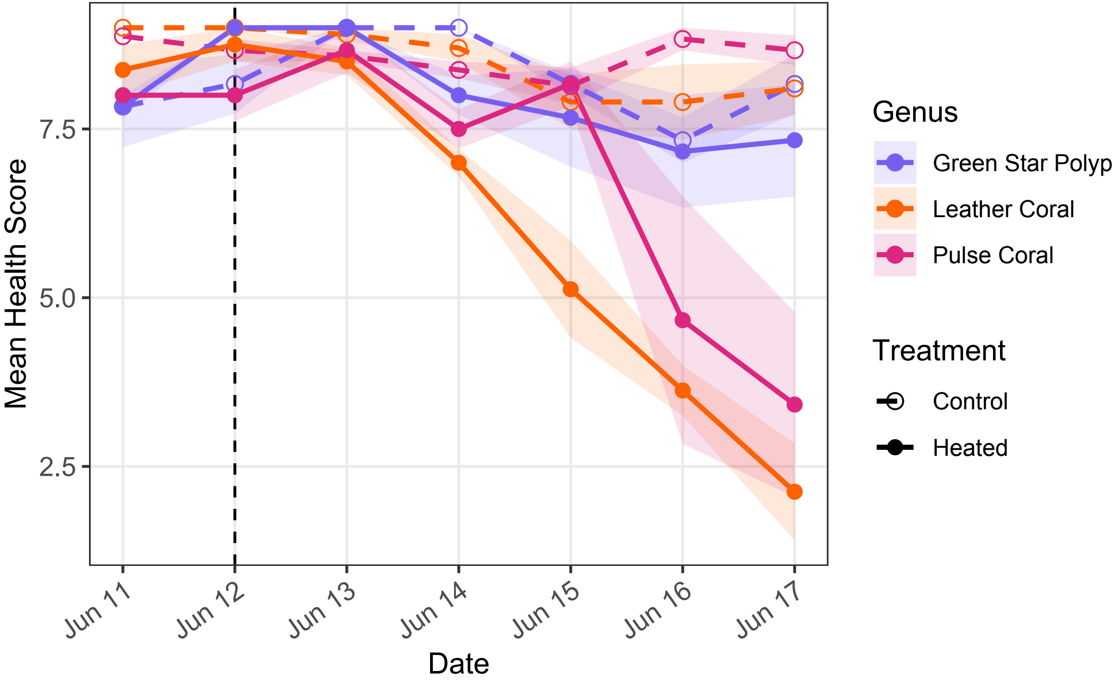

Mean ± SE per Genus. Solid = heated, dashed = control.
Dotted grey line = heated tank temperature (right axis). Score range 1–9.
